# Design, Synthesis, and Antifungal Activity of Novel 1,2,4-Triazolo[4,3-*c*]trifluoromethylpyrimidine Derivatives Bearing the Thioether Moiety

**DOI:** 10.3389/fchem.2022.939644

**Published:** 2022-07-19

**Authors:** Chunyi Liu, Qiang Fei, Nianjuan Pan, Wenneng Wu

**Affiliations:** Food and Pharmaceutical Engineering Institute, Guiyang University, Guiyang, China

**Keywords:** pyrimidine, synthesis, fungicidal activity, 1, 2, 4-triazol, *Botrytis cinerea*

## Abstract

Crop disease caused by fungi seriously affected food security and economic development. Inspired by the utilization of fungicide containing 1,2,4-triazole and trifluoromethylpyrimidine, a novel series of 1,2,4-triazolo[4,3-*c*]trifluoromethylpyrimidine derivatives bearing the thioether moiety were synthesized. Meanwhile, the antifungal activities of the title compounds were evaluated and most compounds exhibited obvious antifungal activities against cucumber *Botrytis cinerea*, strawberry *Botrytis cinerea*, tobacco *Botrytis cinerea*, blueberry *Botrytis cinerea*, *Phytophthora infestans*, and *Pyricularia oryzae* Cav. Among the compounds, 4, 5h, 5o, and 5r showed significant antifungal activities against three of the four *Botrytis cinerea*, which indicated the potential to become the leading structures or candidates for resistance to *Botrytis cinerea*.

## 1 Introduction

Crop disease caused by fungi seriously affected food security and economic development (O’Brien, 2017; Yang et al., 2021). The application of existing antifungal agents was limited, due to the high resistance and security caused by fungicide abuse (Wei et al., 2021). It is increasingly urgent to develop new antimicrobial agents with high antimicrobial performances and good environmental friendliness.

**FIGURE 1:**
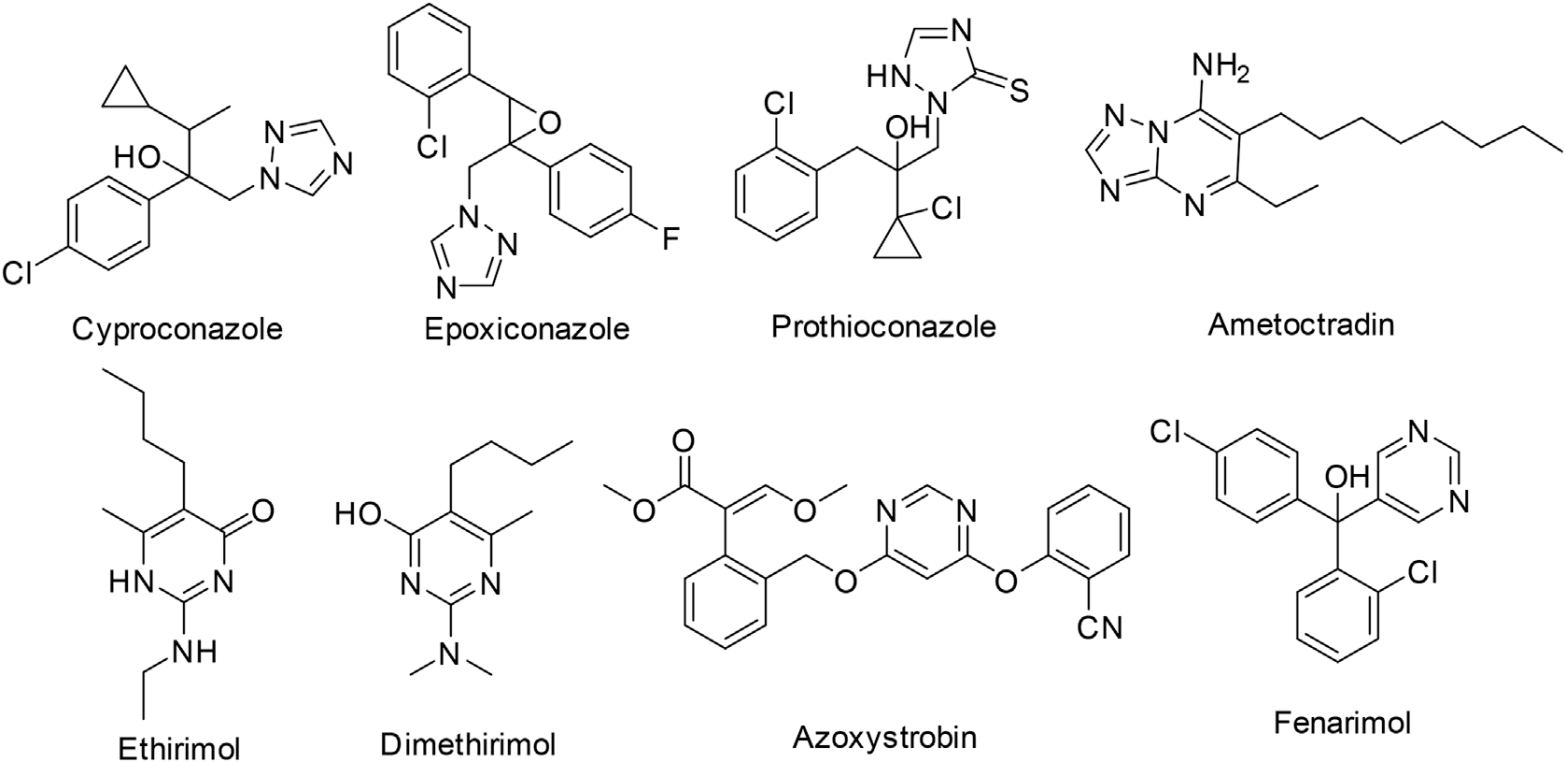
The structure of the sterilizing agent and triamcinolone.

Triazole fungicides are vital five-membered nitrogen-containing heterocycles, which are one of the largest categories of fungicides in the world, and they are widely used in agriculture field. The listed triazole fungicides include Cyproconazole, Epoxiconazole, and Prothioconazole (Figure 1). And there are also triazolopyrimidine fungicides ametoctradin. 1,2,4-Triazole derivatives have been extensively applied in antifungal (Sun et al., 2021), antitumor (Abdelrehim, 2021), antibacterial (Pathak et al., 2021), antiviral (Shao et al., 2021), herbicidal (Yang et al., 2022), and insecticidal (Fan et al., 2019) agents.

Pyrimidine is an essential part of DNA and RNA in living system (Kumar and Narasimhan, 2018). Pyrimidine derivatives exhibited diverse biological properties, such as antineoplastic (Kesari et al., 2021), anti-inflammatory (Abdel-Aziz et al., 2021), antiviral (Abu-Zaied et al., 2021), and antimicrobial (El-mahdy and Farouk, 2021) activities. In addition, fused pyrimidine systems, such as pyrrolo [3,2-*d*]pyrimidine and 1,2,4-triazolo[1,5-*a*]pyrimidine, also possess extensive biological activities (Cawrse et al., 2018; Wang et al., 2019; Baillache and Unciti-Broceta, 2020; Basyouni et al., 2021; Wang R.-X. et al., 2021; Fayed et al., 2022). For example, Abulkhair (El-Shershaby et al., 2021) developed a set of triazoloquinazoline derivatives and screened the most potential compound against four human cancer cell lines. Since the invention of ethirimol, a series of pyrimidine fungicides were commercialized including ethirimol, dimethirimol, azoxystrobin, and fenarimol (Figure 1).

Moreover thioether is generally recognized as a linking structure which is able to reduce the lipophilicity. Meanwhile, the linker is beneficial to enhance the drug likeness of bioactive molecules (Ding et al., 2021).

**FIGURE 2:**
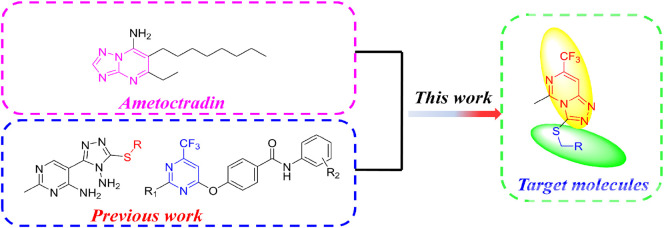
Design of the target molecules.

Based on the aforementioned consideration, we constructed a fused pyrimidine structure similar to ametoctradin. Furthermore, referring our previous works (Wu W. et al., 2019; Yu et al., 2021), trifluoromethyl was introduced into the pyrimidine scaffold and thioether linker was promoted. Finally we designed and synthesized a novel series of thio-1,2,4-triazolo[4,3-*c*]pyrimidine derivatives on account of the molecular hybridization strategy (Figure 2).

## 2 Materials and Methods

### 2.1 Chemistry

All solvents were dried by standard methods in advance and distilled before use. The melting points of the products were determined on a XT-4 binocular microscope (Beijing Tech Instrument Co., China). ^1^H NMR and ^13^C NMR (solvent DMSO-*d*_6_) spectral analyses were performed on a Bruker Avance NEO 600 NMR spectrometer (^1^H, 600 MHz; ^13^C, 150 MHz) at room temperature. TMS was used as an internal standard. Mass spectrometry (MS) data were obtained on a Thermo Scientific Q Exactive Focus instrument. The following abbreviations were used to label chemical shift multiplicities: s = singlet, d = doublet, t = triplet, and m = multiplet. Analytical TLC was performed on silica gel GF-254.

### 2.2 General Procedure for the Preparation of Intermediates 1–2

Intermediates 1 and 2 were synthesized by the methods of our previous work (Wu W.-N. et al., 2019).

### 2.3 General Procedure for the Preparation of Intermediates 3

2-Methyl-4-chloro-6-trifluoromethylpyrimidine (20 mmol) and absolute ethanol (50 ml) were weighed, added into a three-necked flask, and stirred under ice bath conditions, and hydrazine hydrate (30 mmol) was added dropwise slowly to the reaction system; after the addition was completed, the reaction was carried out in an ice bath, and the reaction was followed by TLC until the raw material was completed. Then water was added to obtain a yellow solid. The solid was purified by column chromatography, eluting with petroleum ether: ethyl acetate 20:1, which gave a pale yellow solid.

4-Hydrazinyl-2-methyl-6-(trifluoromethyl)pyrimidine (3): Pale yellow solid; yield 40.6%; m.p.113.4–114.8°C; ^1^H NMR (600 MHz, DMSO-*d*_*6*_) δ 8.45(s, 1H, pyrimidine-H), 7.14(s, 1H, pyrimidine-NH-), 4.60(s, 1H, -NH_2_), 2.86(s, 3H, -CH_3_).

### 2.4 General Procedure for the Preparation of Intermediates 4

Intermediate 3 (50 mmol) and triethylamine (75 mmol) were placed in a three-necked flask of 250 ml, anhydrous ethanol was added to dissolve. CS_2_ (75 mmol) was slowly added dropwise, stirred for 30 min, and then heated to reflux for 3 h; the reaction was detected by TLC. After the reaction was completed, the solvent was rotary-evaporated and water was added to suction filtration to remove insoluble matter. The filtrate was adjusted to pH 3 with 10% hydrochloric acid, placed in a refrigerator, and left to stand overnight, suction-filtered, and dried to obtain intermediate 4.

5-Methyl-7-(trifluoromethyl)-[1,2,4]triazolo [4,3-c]pyrimidine-3-thiol (4) White solid; yield 53.4%; m.p.143.7–144.2°C; ^1^H NMR (600 MHz, DMSO-*d*_*6*_) δ 8.42(s, 1H, pyrimidine-H), 2.91 (s, 3H, CH_3_-); ^13^C NMR (150 MHz, DMSO-*d*_*6*_) δ 165.31, 152.96, 152.82, 142.23(q, *J* = 35.1 Hz), 142.23(q, *J* = 272.25 Hz), 108.16, 20.11; MS (ESI) m/z: 233.1 ([M-H]^-^).

### 2.5 General Procedure for the Preparation of the Target Compounds 5a-5s

Intermediate 4 (10 mmol) was added into a triethylamine (15 mmol) aqueous solution, and after stirring at room temperature for 0.5 h, benzyl chloride (11 mmol) with different substituent was added, the reaction was carried out at room temperature, and the reaction was detected by TLC. After completion, the system was filtered to obtain the solid mixture. The mixture was purified by column chromatography to obtain the target compounds 5a–5s.

*3-*[(*2,4-dichlorobenzyl*)*thio*]*-5-methyl-7-(trifluoromethyl)-*[*1,2,4*]*triazolo*[*4,3-c*]*pyrimidine* (5q): White solid; yield 69.49%; m.p.72.1–74.6°C; ^1^H NMR (600 MHz, DMSO-*d*_6_) δ 8.32(s, 1H, Pyrimidine), 7.69(d, 1H, *J* = 7.8 Hz), 7.67(d, 1H, *J* = 1.8 Hz), 7.34(dd, 1H, *J*_*1*_ = 1.8 H, *J*_*2*_ = 6.6 Hz), 4.64(s, 2H, SCH_2_), 2.93(s, 3H, CH_3_); ^13^C NMR (150 MHz, DMSO-*d*_6_) δ 167.00, 152.58, 152.23, 141.98 (q, *J* = 35.5 Hz), 134.81, 134.32, 133.62, 133.27, 129.44, 127.95, 122.44 (q, *J* = 271.5Hz), 109.41, 32.75, 20.11; MS (ESI) m/z: 393.0([M + H]^+^), 415.0 ([M + Na]^+^).

### 2.6 *In vitro* Antifungal Activity Test

The mycelial growth rates method (Du et al., 2021; Wang S. et al., 2021) was selected to evaluate the antifungal activities of the compounds 5a–5r against six phytopathogenic fungi, including cucumber *Botrytis cinerea*, tobacco *Botrytis cinerea*, blueberry *Botrytis cinerea*, *Phytophthora infestans*, strawberry *Botrytis cinerea*, and *Pyricularia oryzae* Cav. The tested compounds were dissolved in 0.5 ml dimethyl formamide (DMF) and then added 9.5 ml sterile water to prepare the test liquids. The test liquids were poured into 90 ml potato dextrose agar (PDA) to maintain the concentrations of the compounds, 50 *μ*g/ml. Each treatment was replicated three times. The inoculated plates were fostered at 25 ± 1°C for 3–4 days. Tebuconazole and pyraclostrobin were acted as positive controls. The cross method was utilized to measure the diameter of the mycelium. The inhibition rate *I* (%) was calculated through the following formula, where *C* (cm) represents the average diameter of fungi growth on untreated PDA and *T* (cm) represents the average diameter of fungi on treated PDA.

I(%)=[(C−T)/(C−0.4)]×100

## 3 Results and Discussion

### 3.1 Chemistry

**SCHEME 1:**
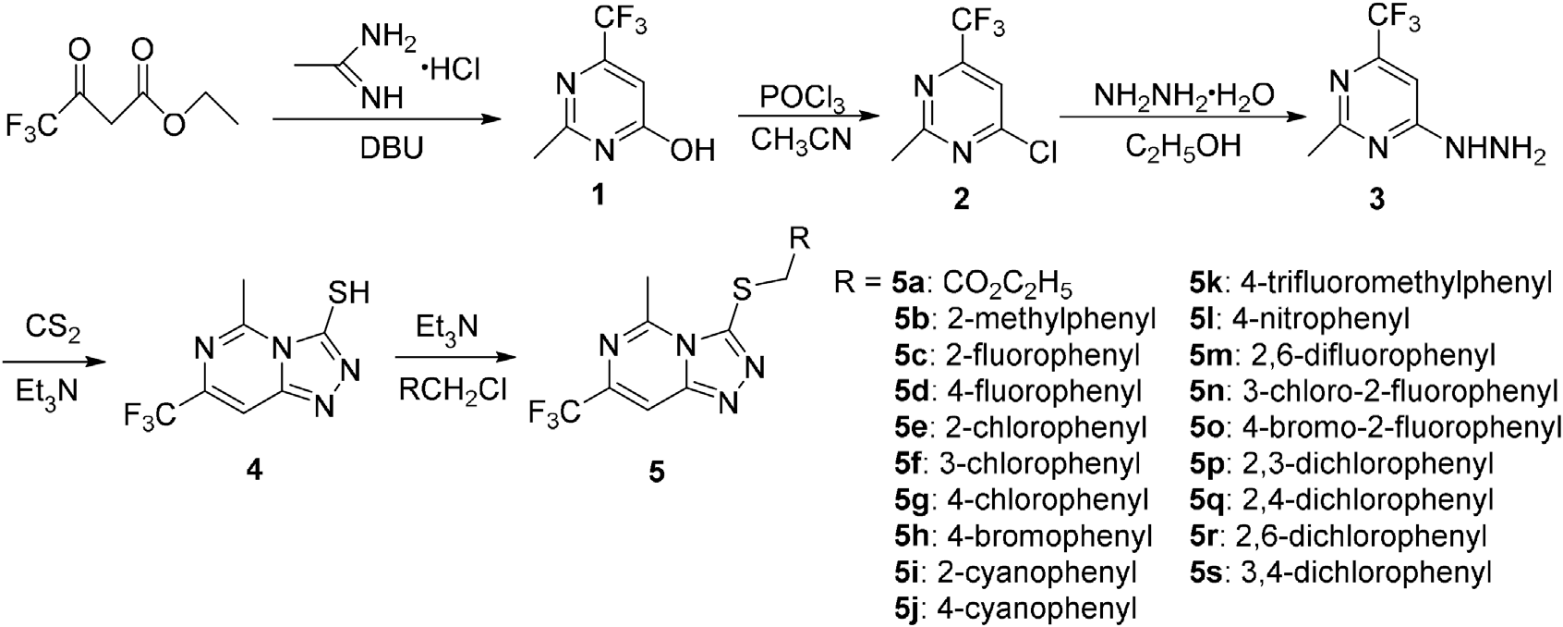
Synthetic route for 5a-5s.

The general synthetic route for the target compounds 5a–5s is depicted in Scheme 1. Acetamidine hydrochloride and ethyl trifluoroacetoacetate were used as raw materials. The target compounds 5a–5s were synthesized in five steps, including cyclization, chlorination, hydrazinolysis, cyclization, and thioetherification. The synthesized compounds were confirmed by ^1^H NMR, ^13^C NMR, and mass spectrometry.

In the ^1^H NMR data of compound 5q, a singlet appeared at 8.32 ppm indicated the presence of CH proton of the 8-trifluoromethylpyrimidine. A dd peak presented at 7.34 ppm signified the presence of CH proton in 3-phenyl. A singlet appeared at 4.64 ppm indicated the presence of CH of -SCH_2_- group. The singlet at 2.93 ppm indicated the CH_3_ proton in pyrimidine ring. Meanwhile, in the ^13^C NMR data of compound 5q, two quartets at 141.98 and 122.44 ppm indicated the presence of -CF_3_ in the pyrimidine fragment. Signals at 32.75 and 20.11 ppm indicated the presence of carbon in the -SCH_2_- and CH_3_. In addition, compound 5q was further confirmed by MS data with the ([M + H]^+^) and [M + Na]^+^peaks.

### 3.2 Antifungal Activity Test *in vitro*

The antifungal activity of key intermediate 4 and target compounds 5a–5s was evaluated through the mycelial growth rates method. Most compounds exhibited some activity towards *Botrytis cinerea* (Table 1). As cucumber *Botrytis cinerea*, the inhibition rates of compounds 4, 5b, 5f, 5h, 5m, 5n, 5o, and 5r were 75.86, 77.78, 71.97, 72.31, 75.63, 76.58, 80.38, and 73.57%, respectively. Moreover, compounds 4, 5g, 5h, 5i, 5j, 5k, 5l, 5o, 5p, 5q, 5r, and 5s showed good antifungal activities against strawberry *Botrytis cinerea* and, the inhibition rates were 82.68, 72.89, 74.37, 76.35, 77.85, 77.32, 76.66, 75.31, 70.60, 71.52, 79.85, and 73.75%, respectively. In particular, four compounds 4, 5h, 5o, and 5r presented obvious activity to three of the four *Botrytis cinerea*. This also indicated the potential of these four compounds as leading structures or candidates against *Botrytis cinerea*.

**TABLE 1:** The antifungal activities of the compounds 4 and 5a–5s against plant pathogens of cucumber *Botrytis cinerea*, strawberry *Botrytis cinerea*, tobacco *Botrytis cinerea*, blueberry *Botrytis cinerea*, *Phytophthora infestans,* and *Pyricularia oryzae* Cav*. in vitro* at 50 *μ*g/ml.

Compounds	Inhibition Rate (%)					
	Cucumber *Botrytis cinerea*	Tobacco *Botrytis cinerea*	Blueberry botrytis *cinerea*	*Phytophthora infestans*	Strawberry *Botrytis cinerea*	*Pyricularia oryzae* Cav*.*
4	75.86 ± 1.80	64.35 ± 2.31	58.68 ± 2.15	45.62 ± 1.12	82.68 ± 1.69	50.34 ± 1.26
5a	61.36 ± 1.56	36.66 ± 1.42	45.28 ± 2.61	16.27 ± 2.64	66.02 ± 1.27	26.85 ± 1.94
5b	77.78 ± 1.35	35.69 ± 1.73	57.65 ± 1.09	24.40 ± 1.17	65.05 ± 2.60	37.65 ± 1.36
5c	61.01 ± 2.47	23.79 ± 2.28	57.01 ± 1.69	15.96 ± 1.05	47.57 ± 1.19	33.65 ± 1.14
5d	55.80 ± 3.12	34.07 ± 1.80	43.33 ± 1.07	20.78 ± 3.38	63.75 ± 1.44	23.46 ± 2.26
5e	45.46 ± 2.59	35.37 ± 1.29	44.30 ± 2.67	13.25 ± 2.07	69.57 ± 1.13	25.61 ± 1.57
5f	71.97 ± 1.35	35.65 ± 1.16	54.72 ± 1.18	26.50 ± 1.18	60.53 ± 3.03	30.56 ± 1.63
5g	62.38 ± 3.05	48.54 ± 2.32	57.34 ± 2.68	17.46 ± 1.56	72.89 ± 1.04	26.24 ± 1.81
5h	72.31 ± 2.05	38.26 ± 1.34	60.23 ± 2.26	23.51 ± 2.13	74.37 ± 3.75	36.18 ± 3.80
5i	53.29 ± 1.84	24.75 ± 2.35	50.17 ± 1.08	12.94 ± 1.29	76.35 ± 3.16	32.41 ± 2.46
5j	65.15 ± 1.61	34.40 ± 1.25	56.68 ± 2.01	20.48 ± 1.08	77.85 ± 3.28	31.80 ± 3.08
5k	67.47 ± 1.64	38.26 ± 3.18	52.21 ± 1.64	15.96 ± 3.66	77.32 ± 3.46	25.93 ± 2.20
5l	53.03 ± 3.01	28.28 ± 2.25	47.24 ± 1.98	15.96 ± 2.51	76.66 ± 3.32	29.31 ± 2.71
5m	75.63 ± 2.17	30.85 ± 1.10	65.04 ± 1.15	21.62 ± 2.11	63.19 ± 2.65	34.37 ± 2.19
5n	76.58 ± 2.26	39.37 ± 1.90	59.70 ± 2.38	31.26 ± 1.24	68.71 ± 2.81	38.22 ± 3.07
5o	80.38 ± 2.41	46.93 ± 2.34	64.12 ± 1.10	38.60 ± 1.87	75.31 ± 1.78	46.97 ± 1.68
5p	53.03 ± 1.95	29.58 ± 1.17	42.35 ± 1.84	14.15 ± 1.39	70.60 ± 1.28	27.46 ± 2.49
5q	63.89 ± 1.15	72.99 ± 3.09	42.02 ± 1.20	19.26 ± 1.35	71.52 ± 1.69	26.54 ± 3.05
5r	73.57 ± 2.94	78.68 ± 2.31	46.12 ± 3.84	23.65 ± 3.12	79.85 ± 2.87	30.26 ± 2.18
5s	55.80 ± 1.68	33.11 ± 1.49	33.56 ± 3.37	17.46 ± 3.79	73.75 ± 1.34	27.46 ± 2.14
Tebuconazole	100.00 ± 1.37	100.00 ± 1.32	100.00 ± 2.23	100.00 ± 1.17	100.00 ± 1.06	81.25 ± 2.28
Pyraclostrobin	100.00 ± 1.13	100.00 ± 2.24	100.00 ± 1.12	100.00 ± 2.08	100.00 ± 2.26	83.34 ± 1.18

Further structure–activity relationship analysis signified that the introduction of halogen atom could improve the antifungal activities against *Botrytis cinerea*. Especially for strawberry *Botrytis cinerea*, it was more obvious. Moreover, the introduction of electron withdrawing group, such as -CN, -CF_3_, and -NO_2_, could increase the activity of compounds to a certain extent. For example, compounds 5i, 5j, 5k, and 5l appeared a good activity against strawberry *Botrytis cinerea*. In addition, most compounds represented ordinary activities towards *Phytophthora infestans and Pyricularia oryzae* Cav*.*, which indicated these compounds were not favor to the inhibition of these two fungi.

## 4 Conclusion

In summary, a novel series of 1,2,4-triazolo[4,3-*c*]trifluoromethylpyrimidine derivatives bearing the thioether moiety were designed, synthesized, and characterized. The antifungal activities of the target compounds were also evaluated. Most compounds exhibited obvious antifungal activities against cucumber *Botrytis cinerea*, strawberry *Botrytis cinerea*, tobacco *Botrytis cinerea*, blueberry *Botrytis cinerea*, *Phytophthora infestans* and *Pyricularia oryzae* Cav*.* Notably, four compounds 4, 5h, 5o, and 5r were selected and showed significant antifungal activities against three of the four *Botrytis cinerea*. This indicated that these compounds are expected to become the leading structures or candidates for resistance to *Botrytis cinerea*.

## Data Availability

The datasets presented in this study can be found in online repositories. The names of the repository/repositories and accession number(s) can be found below: Cambridge Crystallographic Data Centre, 2189490.
