# Supplementary material for: Design, Synthesis, and Antifungal Activity of Novel 1,2,4-Triazolo[4,3-c]trifluoromethylpyrimidine Derivatives Bearing the Thioether Moiety
Source: Front Chem. 2022 Jul 19;10:939644. doi: 10.3389/fchem.2022.939644 (PMC9344801; doi:10.3389/fchem.2022.939644)
Supplement: Supplementary file 1 [file DataSheet1.doc]

Supporting Information

**Design, synthesis, crystal structure characterization and antifungal activity of novel 1,2,4-triazolo[4,3-*c*]trifluoromethylpyrimidine derivatives bearing the thioether moiety**

Chunyi Liu^#^, Qiang Fei^#^*, Nianjuan Pan, Yanbi Luo**,** Wenneng Wu

Food and Pharmaceutical Engineering Institute, Guiyang University, Guiyang, China

*Corresponding author:

fqorganic@163.com(Qiang Fei)

**1. Synthesis and characterization**

*ethyl 2-((5-methyl-7-(trifluoromethyl)-[1,2,4]triazolo[4,3-c]pyrimidin-3-yl)thio)acetate* (**5a**): White solid; yield 45.31%; m.p.73.2-74.2 °C; ^1^H NMR (600 MHz, DMSO-*d*_6_) δ 8.32(s, 1H, Pyrimidine), 4.22(s, 2H, SCH_2_), 4.17 (q, 2H, J=7.2 Hz), 2.90(s, 3H, CH_3_), 1.23 (t, 3H, *J* = 7.2 Hz); ^13^C NMR (150 MHz, DMSO-*d*_6_) δ 168.77, 167.16, 152.44, 152.17, 141.98(q, *J* = 35.5 Hz), 122.43(q, *J* = 271.95Hz), 109.38, 61.78, 33.56, 19.97, 14.50; MS (ESI) m/z: 343.0 ([M+Na]^+^).

*5-methyl-3-((2-methylbenzyl)thio)-7-(trifluoromethyl)-[1,2,4]triazolo[4,3-c]pyrimidine* (**5b**): Pale yellow solid; yield 65.42%; m.p.66.5-66.8 °C; ^1^H NMR (600 MHz, DMSO-*d*_6_) δ 8.31 (t, 1H, *J* = 9.5 Hz), 7.46 (s, 1H), 7.53 (m, 3H), 4.59 (s, 2H), 2.93(s, 3H), 2.42 (s, 3H); ^13^C NMR (150 MHz, DMSO-*d*_6_) δ 167.69, 152.50, 152.13, 141.92 (q, *J* = 35.1 Hz), 137.14, 134.93, 130.81, 130.45, 128.30, 126.53, 122.46(q, *J* = 271.95Hz), 107.31,33.58, 20.07, 19.27; MS (ESI) m/z: 361.0 ([M+Na]^+^).

*5-methyl-3-((2-fluorobenzyl)thio)-7-(trifluoromethyl)-[1,2,4]triazolo[4,3-c]pyrimidine* **(5**c): Pale yellow solid; yield 55.86%; m.p.80.4-81.2 °C; ^1^H NMR (600 MHz, DMSO-*d_6_*) δ 8.31(s, 1H, Pyrimidine), 7.51(t, 1H, *J* = 7.8 Hz), 7.35(q, 1H, *J* = 7.8 Hz), 7.23(t, 1H, *J* = 9.6 Hz), 7.17(t, 1H, *J* = 7.8 Hz), 4.59(s, 2H, SCH_2_), 2.92(s, CH_3_); ^13^C NMR (150 MHz, DMSO-*d*_6_) δ 167.32, 160.12, 152.55, 152.16, 142.03(q, *J* = 35.25 Hz), 131.89, 130.29, 124.93, 124.64, 124.54, 122.44(q, *J* = 271.65Hz), 115.90, 107.26, 28.80, 20.00; MS (ESI) m/z: 343.1([M+H]^+^), 365.0 ([M+Na]^+^).

*5-methyl-3-((4-fluorobenzyl)thio)-7-(trifluoromethyl)-[1,2,4]triazolo[4,3-c]pyrimidine* (**5d**): Pale yellow solid; yield 68.25%; m.p.82.5-82.9 °C; ^1^H NMR (600 MHz, DMSO-*d*_6_) δ 8.26(s, 1H, Pyrimidine),7.51(dd, 2H, *J_1_* = 5.4 Hz, *J_2_* = 8.4 Hz), (t, 2H, *J* = 8.4 Hz), 4.56(s, 2H, SCH_2_), 2.92(s, CH_3_); ^13^C NMR (150 MHz, DMSO-*d*_6_) δ 167.58, 162.69, 161.08, 152.46, 152.15, 142.03(q, *J*=35.25 Hz), 134.14, 131.55, 122.40(q, *J* = 271.8Hz), 115.72, 115.58, 107.22, 34.30, 20.01; MS (ESI) m/z: 343.0([M+H]^+^), 365.1 ([M+Na]^+^).

*5-methyl-3-((2-chlorobenzyl)thio)-7-(trifluoromethyl)-[1,2,4]triazolo[4,3-c]pyrimidine* (**5e**): Pale yellow solid; yield 68.25%; m.p.66.8-68.1 °C; ^1^H NMR (600 MHz, DMSO-*d*_6_) δ 8.30(s, 1H, Pyrimidine), 7.60(s, 1H), 7.51(d, 1H, *J* = 7.2 Hz), 7.36-7.30(m, 2H), 4.56(s, 2H, SCH_2_), 2.92(s, CH_3_); ^13^C NMR (150 MHz, DMSO-*d*_6_) δ 167.41, 152.51, 152.11, 142.03(q, *J* = 35.4 Hz), 140.62, 133.33, 130.73, 129.41, 128.22, 127.78, 122.43(q, *J* = 271.65Hz), 115.90, 107.31, 34.39, 20.03; MS (ESI) m/z: 359.1([M+H]^+^), 381.0 ([M+Na]^+^).

*5-methyl-3-((3-chlorobenzyl)thio)-7-(trifluoromethyl)-[1,2,4]triazolo[4,3-c]pyrimidine* (**5f**): Pale yellow solid; yield 68.25%; m.p.66.8-68.1 °C; ^1^H NMR (600 MHz, DMSO-*d*_6_) δ 8.30(s, 1H, Pyrimidine), 7.60(s, 1H), 7.51(d, 1H, *J* = 7.2 Hz), 7.36-7.30(m, 2H), 4.56(s, 2H, SCH_2_), 2.92(s, CH_3_); ^13^C NMR (150 MHz, DMSO-*d*_6_) δ 167.41, 152.51, 152.11, 142.03(q, *J* = 35.4 Hz), 140.62, 133.33, 130.73, 129.41, 128.22, 127.78, 122.43(q, *J* = 271.65Hz), 115.90, 107.31, 34.39, 20.03; MS (ESI) m/z: 359.1([M+H]^+^), 381.0 ([M+Na]^+^).

*5-methyl-3-((4-chlorobenzyl)thio)-7-(trifluoromethyl)-[1,2,4]triazolo[4,3-c]pyrimidine* (**5g**): Pale yellow solid; yield 68.25%; m.p.72.3-73.1°C; ^1^H NMR (600 MHz, DMSO-*d*_6_) δ 8.28(s, 1H, Pyrimidine), 7.51(d, 1H, *J* = 8.4 Hz), 7.35(d, 1H, *J* = 8.4 Hz), 4.54(s, 2H, SCH_2_), 2.92(s, CH_3_); ^13^C NMR (150 MHz, DMSO-*d*_6_) δ 167.53, 152.48, 152.11, 142.03(q, *J* = 35.4 Hz), 137.07, 132.48, 131.32, 128.79, 122.42(q, *J* = 271.8Hz), 107.26, 34.39, 20.01; MS (ESI) m/z: 359.1([M+H]^+^), 381.0 ([M+Na]^+^).

*5-methyl-3-((4-bromobenzyl)thio)-7-(trifluoromethyl)-[1,2,4]triazolo[4,3-c]pyrimidine* (**5h**): Pale yellow solid; yield 86.7%; m.p.67.9-68.2 °C; ^1^H NMR (600 MHz, DMSO-*d*_6_) δ 8.28(s, 1H, Pyrimidine), 7.51(d, 1H, *J* = 8.4 Hz), 7.35(d, 1H, *J* = 8.4 Hz), 4.54(s, 2H, SCH_2_), 2.92(s, CH_3_); ^13^C NMR (150 MHz, DMSO-*d*_6_) δ 167.53, 152.48, 152.11, 142.03(q, *J* = 35.4 Hz), 137.07, 132.48, 131.32, 128.79, 122.42(q, *J* = 271.8Hz), 107.26, 34.39, 20.01; MS (ESI) m/z: 400.9([M-H]^-^).

*2-(((5-methyl-7-(trifluoromethyl)-[1,2,4]triazolo[4,3-c]pyrimidin-3-yl)thio)methyl)benzo-nitrile* (**5i**): White solid; yield 71.56%; m.p.103.1-105.9 °C; ^1^H NMR (600 MHz, DMSO-d_6_) δ 8.30(s, 1H, Pyrimidine), 7.87(d, 1H, J=7.2 Hz), 7.82(d, 1H, J=7.8 Hz), 7.68(t, 1H, J=7.8 Hz), 7.49(t, 1H, J=7.8 Hz), 4.73(s, 2H, SCH_2_), 2.93(s, CH_3_); ^13^C NMR (150 MHz, DMSO-d_6_) δ 166.74, 152.62, 152.25, 141.96(q, *J*=34.95 Hz), 141.32, 133.82, 133.53, 130.98, 128.91, 122.42(q, *J*=272.1 Hz), 117.89, 112.49, 107.43, 33.53, 20.09; MS (ESI) m/z: 372.0 ([M+Na]^+^).

*4-(((5-methyl-7-(trifluoromethyl)-[1,2,4]triazolo[4,3-c]pyrimidin-3-yl)thio)methyl)benzo-nitrile* (**5j**): White solid; yield 64.56%; m.p.116.0-117.3 °C; ^1^H NMR (600 MHz, DMSO-*d_6_*) δ 8.31(s, 1H, pyrimidine-H), 7.80(d, 2H, *J* = 8.4 Hz), 7.72(d, 2H, *J* = 8.4 Hz), 4.64 (s, 2H, -CH_2_S-), 2.92 (s, 3H, CH_3_-); ^13^C NMR (150 MHz, DMSO-*d_6_*) δ 167.15, 152.543, 152.19, 144.18, 141.96(q, *J* = 34.8 Hz), 132.79, 130.53, , 122.44(q, *J* = 271.95 Hz), 119.17, 110.55, 107.36, 34.60, 20.07; MS (ESI) m/z: 372.0 ([M+Na]^+^).

*5-methyl-7-(trifluoromethyl)-3-((4-(trifluoromethyl)benzyl)thio)-[1,2,4]triazolo[4,3-c]pyrimidine* (**5k**): White solid; yield 62.72%; m.p.89.5-89.9 °C; ^1^H NMR (600 MHz, DMSO-*d_6_*) δ 8.29(s, 1H, pyrimidine-H), 7.75(q, *J* = 8.4 Hz), 7.63(q, *J* = 8.4 Hz), 4.65 (s, 2H, -CH_2_S-), 2.92 (s, 3H, CH_3_-); ^13^C NMR (150 MHz, DMSO-*d_6_*) δ 167.29, 152.53, 152.16, 141.97(q, *J* = 35.1 Hz), 143.11, 131.37, 130.30, 128.81, 128.48(q, *J* = 31.5 Hz), 125.68, 142.23(q, *J* = 274.2 Hz), 122.42(q, *J* = 271.8 Hz), 107.33, 34.43, 20.03; MS (ESI) m/z: 393.1([M+H]^+^), 415.0 ([M+Na]^+^).

*5-methyl-3-((4-nitrobenzyl)thio)-7-(trifluoromethyl)-[1,2,4]triazolo[4,3-c]pyrimidine* (**5l**): White solid; yield 48.96%; m.p.159.8-160.5°C; ^1^H NMR (600 MHz, DMSO-*d_6_*) δ 8.31(s, 1H, pyrimidine-H), 8.19(d, 2H, *J* = 8.4 Hz), 7.81(d, 2H, *J* = 9.0 Hz), 4.69 (s, 2H, -CH_2_S-), 2.92 (s, 3H, CH_3_-); ^13^C NMR (150 MHz, DMSO-*d_6_*) δ 167.05, 152.56, 152.23, 147.14, 146.40, 141.96(q, *J* = 34.65 Hz), 130.82, 123.98, 122.44(q, *J* = 272.4 Hz), 107.38, 34.60, 20.07; MS (ESI) m/z: 392.0 ([M+Na]^+^).

*3-((2,6-difluorobenzyl)thio)-5-methyl-7-(trifluoromethyl)-[1,2,4]triazolo[4,3-c]pyrimidine* (**5m**): White solid; yield 41.0%; m.p.107.9-109.3°C; ^1^H NMR (600 MHz, DMSO-*d*_6_) δ 8.32(s, 1H, Pyrimidine), 7.69(d, 1H, *J* = 7.8 Hz), 7.59(d, 1H, *J* = 7.8 Hz), 7.34(t, 1H, *J* = 7.8 Hz), 4.58(s, 2H, SCH_2_); ^13^C NMR (150 MHz, DMSO-*d*_6_) δ 167.06, 152.58, 152.21, 142.03(q, *J* = 35.4 Hz), 137.81, 132.51, 131.90, 130.54, 130.37, 128.64, 122.43(q, *J* = 271.8Hz), 109.37, 34.09, 20.05; MS (ESI) m/z: 383.0 ([M+Na]^+^).

*3-((3-chloro-2-fluorobenzyl)thio)-5-methyl-7-(trifluoromethyl)-[1,2,4]triazolo[4,3-c]pyrimidine* (**5n**): Pale yellow solid; yield 81.4%; m.p.68.8-70.2°C; ^1^H NMR (600 MHz, DMSO-*d*_6_) δ 8.32(s, 1H, Pyrimidine), 7.69(d, 1H, *J* = 7.8 Hz), 7.59(d, 1H, *J* = 7.8 Hz), 7.34(t, 1H, *J* = 7.8 Hz), 4.58(s, 2H, SCH_2_); ^13^C NMR (150 MHz, DMSO-*d*_6_) δ 167.06, 152.58, 152.21, 142.03(q, *J* = 35.4 Hz), 137.81, 132.51, 131.90, 130.54, 130.37, 128.64, 122.43(q, *J* = 271.8Hz), 109.37, 34.09, 20.05; MS (ESI) m/z: 375.0([M-H]^-^).

*3-((4-bromo-2-fluorobenzyl)thio)-5-methyl-7-(trifluoromethyl)-[1,2,4]triazolo[4,3-c]pyrimidine* (**5o**): Pale yellow solid; yield 83.7%; m.p.68.8-70.2°C; ^1^H NMR (600 MHz, DMSO-*d*_6_) δ 8.32(s, 1H, Pyrimidine), 7.69(d, 1H, *J* = 7.8 Hz), 7.59(d, 1H, *J* = 7.8 Hz), 7.34(t, 1H, *J* = 7.8 Hz), 4.58(s, 2H, SCH_2_); ^13^C NMR (150 MHz, DMSO-*d*_6_) δ 167.06, 152.58, 152.21, 142.03(q, *J* = 35.4 Hz), 137.81, 132.51, 131.90, 130.54, 130.37, 128.64, 122.43(q, *J* = 271.8Hz), 109.37, 34.09, 20.05; MS (ESI) m/z: 443.0 ([M+Na]^+^).

*3-((2,3-dichlorobenzyl)thio)-5-methyl-7-(trifluoromethyl)-[1,2,4]triazolo[4,3-c]pyrimidine* (**5p**): White solid; yield 62.34%; m.p.114.2-115.8°C; ^1^H NMR (600 MHz, DMSO-*d*_6_) δ 8.32(s, 1H, Pyrimidine), 7.69(d, 1H, *J* = 7.8 Hz), 7.59(d, 1H, *J* = 7.8 Hz), 7.34(t, 1H, *J* = 7.8 Hz), 4.58(s, 2H, SCH_2_); ^13^C NMR (150 MHz, DMSO-*d*_6_) δ 167.06, 152.58, 152.21, 142.03(q, *J* = 35.4 Hz), 137.81, 132.51, 131.90, 130.54, 130.37, 128.64, 122.43(q, *J* = 271.8Hz), 109.37, 34.09, 20.05; MS (ESI) m/z: 415.0 ([M+Na]^+^).

*3-((2,6-dichlorobenzyl)thio)-5-methyl-7-(trifluoromethyl)-[1,2,4]triazolo[4,3-c]pyrimidine* (**5r**): White solid; yield 75.56%; m.p.103.9-104.7°C; ^1^H NMR (600 MHz, DMSO-*d*_6_) δ 8.32(s, 1H, Pyrimidine), 7.69(d, 1H, *J* = 7.8 Hz), 7.67(d, 1H, *J* = 1.8 Hz), 7.34(dd, 1H, *J_1_* = 1.8 H, *J_2_* = 6.6 Hz), 4.64(s, 2H, SCH_2_), 2.93(s, 3H, CH_3_); ^13^C NMR (150 MHz, DMSO-*d*_6_) δ167.00, 152.58, 152.23, 141.98(q, *J* = 35.5 Hz), 134.81, 134.32, 133.62, 133.27, 129.44, 127.95, 122.44(q, *J* = 271.5Hz), 109.41, 32.75, 20.11; MS (ESI) m/z: 415.0 ([M+Na]^+^).

*3-((3,4-dichlorobenzyl)thio)-5-methyl-7-(trifluoromethyl)-[1,2,4]triazolo[4,3-c]pyrimidine* (**5s**): White solid; yield 75.64%; m.p.106.8-107.4°C; ^1^H NMR (600 MHz, DMSO-*d*_6_) δ 8.31(s, 1H, Pyrimidine), 7.69(d, 1H, *J* = 4.2 Hz), 7.59(d, 1H, *J* = 7.8 Hz), 7.34(dd, 1H, *J_1_* = 1.8 Hz, *J_2_* = 8.4 Hz), 4.55(s, 2H, SCH_2_), 2.92(s, CH_3_); ^13^C NMR (150 MHz, DMSO-*d*_6_) δ 167.22, 152.55, 152.15, 142.03(q, *J* = 35.1 Hz), 139.53, 131.62, 131.25, 131.00, 130.42, 129.92, 122.44(q, *J* = 271.5 Hz), 109.33, 33.85, 20.03; MS (ESI) m/z: 393.0([M+H]^+^).

**2. The crystal data of 5q**

**Table S1. Crystal data of partly of the target compound 5q**

| Compound | | **5q** | | | |
| --- | --- | --- | --- | --- | --- |
| Empirical formula | C_15_H_10_Cl_2_F_3_N_3_S | μ/mm^‑1^ | | | 5.417 |
| Formula weight | 392.21 | F(000) | | | 792.0 |
| Temperature/K | 250.01(10) | Crystal size/mm^3^ | | | 0.15 × 0.12 × 0.1 |
| Crystal system | monoclinic | Radiation | | | Cu Kα (λ = 1.54184) |
| Space group | P2_1_/c | 2Θ range for data collection/° | | | 6.414 to 143.08 |
| a/Å | 11.6567(3) | Index ranges | | -14 ≤ h ≤ 13, -5 ≤ k ≤ 3, -33 ≤ l≤33 | |
| b/Å | 4.80480(10) | Reflections collected | | | 6825 |
| c/Å | 28.1051(7) | Independent reflections | 2942 [R_int_ = 0.0290, R_sigma_ = 0.0388] | | |
| α/° | 90 | Data/restraints/parameters | | | 2942/0/218 |
| β/° | 101.336(2) | Goodness-of-fit on F^2^ | | | 1.106 |
| γ/° | 90 | Final R indexes [I>=2σ (I)] | | | R_1_ = 0.0358, wR_2_ = 0.1000 |
| Volume/Å^3^ | 1543.40(6) | Final R indexes [all data] | | | R_1_ = 0.0374, wR_2_ = 0.1014 |
| Z | 4 | Largest diff. peak/hole / e Å^-3^ | | | 0.28/-0.31 |
| ρ_calc_g/cm^3^ | 1.692 |  | | |  |
